# Gonadotrope-Specific Expression and Regulation of Ovine Follicle Stimulating Hormone Beta: Transgenic and Adenoviral Approaches Using Primary Murine Gonadotropes

**DOI:** 10.1371/journal.pone.0066852

**Published:** 2013-07-18

**Authors:** Jingjing Jia, Farideh Shafiee-Kermani, William L. Miller

**Affiliations:** 1 Mount Sinai School of Medicine, Department of Neurology, New York City, New York, United States of America; 2 Department of Molecular and Structural Biochemistry, North Carolina State University, Raleigh, North Carolina, United States of America; Institut Jacques Monod, France

## Abstract

The beta subunit of follicle stimulating hormone (FSHB) is expressed specifically in pituitary gonadotropes in vertebrates. Transgenic mouse studies have shown that enhancers in the proximal promoter between −172/−1 bp of the ovine *FSHB* gene are required for gonadotrope expression of ovine *FSHB*. These enhancers are associated with regulation by activins and gonadotropin releasing hormone (GnRH). Additional distal promoter sequence between −4741/−750 bp is also required for expression. New transgenic studies presented here focus on this distal region and narrow it to 1116 bp between −1866/−750 bp. In addition, adenoviral constructs were produced to identify these critical distal sequences using purified primary mouse gonadotropes as an in vitro model system. The adenoviral constructs contained −2871 bp, −750 bp or −232 bp of the ovine *FSHB* promoter. They all showed gonadotrope-specific regulation since they were induced only in purified primary gonadotropes by activin A (50 ng/ml) and inhibited by GnRH (100 nM) in the presence of activin (except −232*FSHBLuc*). However, basal expression of all three viral constructs (in the presence of follistatin to block cellular induction by activin) was relatively high in pituitary non-gonadotropes as well as gonadotropes. Thus, gonadotrope-specific regulation associated with the proximal promoter was observed as expected, but the model was blind to distal promoter elements between −2871/−750 necessary for gonadotrope-specific expression of ovine *FSHB* in vivo. The new adenoviral-based *in vitro* technique did detect, however, a novel GnRH response element between −750 bp and −232 bp of the ovine *FSHB* promoter. We conclude that adenoviral-based studies in primary gonadotropes can adequately recognize regulatory elements on the ovine *FSHB* promoter associated with gonadotrope-specific regulation/expression, but that more physiologically based techniques, such as transgenic studies, will be needed to identify sequences between −1866/−750 bp of the ovine *FSHB* promoter that are also required for tissue/cell specific expression in vivo.

## Introduction

It is well known that follicle stimulating hormone (FSH) is important for reproduction in all vertebrates. It is expressed only in pituitary gonadotropes and is secreted primarily through a constitutive pathway [1] so serum FSH usually mirrors intracellular production of FSH. FSH is an α/β heterodimer and since transcription for its beta subunit (FSHB) is rate limiting, *FSHB* transcription has major control over serum FSH and, therefore, affects reproduction directly.

Transcription of *FSHB* appears to be induced primarily by activins which are associated with a Smad response element on the ovine *FSHB* promoter between −172/−158 bp [2]. In addition, there is a Pitx1 site located between −60/−53 bp that is also required for expression [3], [4], [5]. These sequences were first identified using transient expression assays with mutated ovine *FSHB*-promoter-luciferase reporter constructs (o*FSHBLuc*) in LβT2 cells (transformed embryonic mouse gonadotropes). Follow up studies using the same mutated o*FSHBLuc* constructs in transgenic mice showed that without the −172/158 site, only 0.1–1% of ovine *FSHB* expression is observed in gonadotropes in vivo [2]. Therefore, this proximal promoter site is required for gonadotrope-specific expression and is also associated with gene regulation in vivo.

Gonadotropin releasing hormone (GnRH) is also a powerful regulator of gonadotropins, but to date, a specific site has not been clearly identified for positive or negative regulation by GnRH on the ovine *FSHB* promoter [6], [7], [8] and some of its effects on *FSHB* expression may be indirect [9]. In this study, the negative effect of chronic high levels of GnRH on o*FSHBLuc* expression was used to demonstrate gonadotrope-specific regulation by GnRH because it is more dramatic and more easily measured than the stimulatory effect of GnRH which is observed best when GnRH is delivered in a pulsatile manner.

Strong evidence exists for other site(s) being critical for gonadotrope-specific expression of ovine *FSHB* in vivo. These data come from studies that used −4741 bp of ovine *FSHB* promoter to express luciferase (−4147o*FSHBLuc*) in transgenic mice [10]. This transgene expressed high levels of luciferase activity (1–10 million RLU/mg pituitary protein), but no expression was observed when either −215o*FSHBLuc* or −750o*FSHBLuc* were used as transgenes [10]. Therefore, sequences between −4741 bp and −750 bp in the distal promoter are also necessary for ovine *FSHB* expression in vivo. These sequences are currently undefined.

In vitro studies with LβT2 cells seemed promising for defining all sequences needed for regulating and expressing ovine *FSHB* in vivo [11]. In fact, LβT2 cells have been used successfully to define response elements in the proximal promoter of ovine *FSHB* necessary for induction by activin in vivo [2]. The induction of −215, −750, or −4741o*FSHBLuc* in LβT2 cells varies between 4- and 12-fold. These constructs are also expressed in non-gonadotropes. Five cell lines, including NIH3T3 fibroblasts, express them only 20–50% less than LβT2 cells [11]. This is surprising since non-gonadotropes do not express −4741o*FSHBLuc* in vivo. In transgenic mice, for example, −4741o*FSHBLuc* is expressed, at least, 1000–2000x more in pituitary gonadotropes than non-gonadotropes and usually expression in non-gonadotropes is undetectable [10], [12]. Therefore, LβT2 cells have been useful for detecting regulatory sequences in the ovine *FSHB* proximal promoter, but has failed to identify equally important sequences in the distal promoter (−4741/−750 bp) needed for gonadotrope-specific expression in vivo.

Recent transgenic studies in the rat suggest that Prop-1 sites between −850 bp and −750 bp of the distal porcine *FSHB* promoter are critical for expression of porcine FSHB [13], [14], but the ovine *FSHB* promoter has no similar Prop-1 sites. The gene encoding human FSHB needs only −350 bp of 5′ promoter sequences, but absolutely requires distal 3′ sequences between +2138 bp and +3142 bp for transgenic expression in the mouse pituitary [15]. Again, sequences within this region show no obvious homology to ovine promoter sequences. Therefore, none of the transgenic studies completed to date with any *FSHB* gene have helped to define specific sequences between −750 bp and −4741 bp needed for gonadotrope-specific expression of ovine *FSHB*.

The studies reported here used a two-pronged approach for defining novel sites on the ovine *FSHB* promoter required for gonadotrope-specific expression. One was computer-driven and discovered highly conserved sequence patterns in the ovine, porcine and human upstream 5′ promoters. These sequences were considered potential elements that might direct gonadotrope-specific expression, so they were deleted from wild type −4741o*FSHBLuc* and the resulting constructs were used as transgenes to determine if the deleted sequences were important for gonadotrope-specific expression. The second approach used viral infection of primary gonadotropes and pituitary non-gonadotropes with viral o*FSHBLuc* constructs (o*FSHBLuc*-V) since these adult primary cells had a good chance of producing regulatory factors that create *FSHB* expression in gonadotropes or repress it in non-gonadotropes. It was thought that although LβT2 cells are of gonadotrope origin, they are transformed and might not produce one or more of these critical factors. To analyze expression of viral constructs (o*FSHBLuc*-V), we measured basal expression (follistatin was added to inhibit culture-produced activin thereby yielding “basal expression”), maximal expression (activin was added to fully induce expression) and GnRH inhibited expression (GnRH was added along with activin to inhibit its induction [9]).

The studies presented here using adenoviral constructs and primary gonadotropes offer a new in vitro approach for analyzing expression and regulation of FSHB, but it also emphasizes the need for a better model to identify sequences between −1866/−750 bp on the ovine *FSHB* promoter needed for turning on or off *FSHB* expression in gonadotropes and non-gonadotropes, respectively. Such expression may depend on the modification of chromatin-embedded *FSHB* DNA triggered by changes in development by cell-cell interactions including gap junctions or by autocrine/paracrine pituitary factors that cannot be mimicked in vitro.

## Materials and Methods

### Reagents

Recombinant human activin A and follistatin (288) were obtained from R&D Systems (Minneapolis, MN). Gonadotropin releasing hormone (GnRH), oligonucleotide primers for preparing viral constructs, collagenase Type I and Pancreatin were all purchased from Sigma-Aldrich (St Louis, MO). Fugene 6 was purchased from Roche Applied Science (Indianapolis, IN) and restriction enzymes plus luciferase assay kits were from Promega (San Luis Obispo, CA). Integrated DNA Technologies was the source of oligos for PCR. Dulbecco’s modified eagle medium and fetal bovine serum were bought from Invitrogen (Carlsbad, CA). Common reagents such as buffers, growth media and agar were purchased from Fisher Scientific, (Inc. Pittsburgh, PA).

### Generation of Promoter-reporter Plasmids, Transgenes and Transgenic Mice

#### Ethics statement

All transgenic mice were maintained and studied with the approval and oversight of the Institutional Animal Use Committee at the University of North Carolina, Chapel Hill, NC, or North Carolina State University. All transgenic mice were bred and cared for at the Biological Resource Facility of North Carolina State University. The wild type ovine *FSHB* promoter-reporter plasmid (−4741o*FSHBLuc*) was described previously [10]. Briefly, it contained 4741 bp of the ovine *FSHB* promoter plus intron 1 driving expression of a luciferase gene in the GL3 basic vector. This plasmid was used to generate the LO- and LS-o*FSHBLuc* plasmid constructs that contain either −2361 bp or −1866 bp, respectively. Using site directed mutagenesis, a KpnI restriction site was produced at −2361 or −1866 and plasmids were digested and re-ligated to produce LO- or LS-o*FSHBLuc*, respectively.

To generate transgenic mice, the constructs were digested with KpnI and BamHI to release them from the plasmid backbone. The digests were sent to the University of North Carolina for purification and injection into the pronuclei of fertilized B6SJL mouse eggs. Transgenic mice were genotyped as described previously [10].

### Pituitary Cell Cultures

Mice carrying LO- or LS-o*FSHBLuc* transgenes were sacrificed at 7–40 weeks of age using CO_2_ and their pituitaries were collected within 5 min before being dispersed into single cell suspensions as described [10]. Briefly, pituitaries were sliced into small pieces and digested with collagenase type I (Sigma-Aldrich, Woodlands, TX) and Pancreatin (Life Technologies, Inc). The yield was approximately 5×10^5^ cells/pituitary. Cells were then plated in 96-well Primaria tissue culture plates (Becton Dickinson & Co., Franklin Lakes, NJ) at a density of 30,000 cells/well in 50 ul of medium 199 containing 10% charcoal-treated sheep serum plus 100 U/ml penicillin G and 100 ug/ml streptomycin (Sigma-Aldrich, Woodlands, TX) and allowed to attach for 2 days at 37°C under an atmosphere of 95% air: 5% CO_2_ in a humidified chamber before treatment. Cells were treated with hormones at the indicated doses and times as described in the figure legends. Treatments were terminated by lysis in 50 µl of 1× passive lysis buffer (Promega Co., Madison, WI), and 15 µl of each cell lysate was assayed for luciferase activity. All of the experiments were performed at least three times and each treatment was assayed in triplicate or quadruplicate.

### Luciferase Assay

For in vivo experiments, mouse pituitaries or other tissues (0.3–1-mg) were snap-frozen in liquid nitrogen before homogenization in 200 µl of cell lysis reagent (Promega Co., Madison, WI). Cell debris was removed by centrifugation at 10,000×g for 20 sec, and then 10 µl of all cell lysates were immediately assayed for luciferase activity using the luciferase assay system (Promega Co., Madison, WI). Activity was measured for 20 sec using an automated 1420 Victor-Light micro plate luminometer (PerkinElmer, Waltham, MA). Finally, protein was assayed using a Qubit fluorometer (Invitrogen, Carlsbad, CA). For primary pituitary cultures, cells were lysed using 50 µl passive Lysis solution (promega Co., Madison, WI) and 15 ul were analyzed in duplicate using the luciferase assay system.

### Truncated Promoter-luciferase Constructs (Plasmids)

Three plasmids containing different lengths of the ovine *FSHB* promoter controlling luciferase expression were produced as reported previously (−195o*FSHLuc*, −748o*FSHBLuc*, and −2932o*FSHBLuc*) [16]. The shortest promoter construct contained all the sequences associated with regulation by activin/GnRH [7], but should not be expressed in either gonadotropes or non-gonadotropes because it lacks distal promoter sequences [10]. The longest construct contained all proximal and distal sequences required for gonadotrope-specific expression of o*FSHBLuc* constructs in transgenic mice and should be expressed in gonadotropes, but not in non-gonadotropes. The −748o*FSHBLuc* construct is essentially the same as −750o*FSHBLuc* and it is the longest construct we tested in transgenic mice that did not express in any cell type in vivo [10]. The empty pGL3 plasmid did not express any luciferase activity when transfected alone.

### Cell Culture and Transient Transfection

Transformed LβT2 cells were obtained as a kind gift from the laboratory of Dr. Pamela Mellon [17]. Cell culture and transfections with Fugene 6 (Roche Molecular Biochemicals, Basel Switzerland) were described previously [2].

### Gonadotrope Purification from H2Kk Transgenic Mice

Gonadotropes were purified from hemizygous H2Kk transgenic mice that were 7–40 weeks old [12]. The method for dispersing pituitary cells was the same as that outlined above (Pituitary cell culture). After dispersion, gonadotropes were separated from non-gonadotropes with two cycles of purification as previously reported [12]. Non-gonadotropes were used to show expression of o*FSHBLuc*-V constructs in inappropriate cell types so non-gonadotropes were also purified through two cycles to deplete them of as many gonadotropes as possible. All cells were cultured as noted above.

### Adenoviral Constructs and Adenovirus Amplification

Three adenoviral constructs were obtained using Adeno-X™ Expression Systems from Clontech (Mountain View, CA). These adenoviral constructs contained −232o*FSHLuc*, −750o*FSHLuc* and −2871o*FSHBLuc* in a viral context. Two of the constructs (−750o*FSHLuc*-V and −2871o*FSHBLuc*-V) were generated using the Adeno-X™ Expression System II. These truncated promoters plus intron 1 connected to the luciferase gene were amplified using Advantage-HF 2 PCR Kit (Clontech, Mountain View, CA) with the following primers:

5′-acggtaccggacatatgaatgcatcagctagcaaaca -3′(−2871 forward),

5′-gaattcccgggcatatggaattacacggcatctttc-3′(−2871 & −750 reverse),

5′-acggtaccggacatatgcatggagctcttagtctact-3′ (−750 forward).

Then the amplified sequences were inserted into the promoterless pDNR-1r donor vector (System II) using the In-Fusion Advantage PCR Cloning Kit (Clontech, Mountain View, CA), and target sequences on the donor vector were put into the adenoviral backbone by Cre recombinase (System II). The insertion was confirmed by PCR analysis using the Adeno-X™ PCR screening primer set (Clontech, Mountain View, CA).

The third adenoviral construct (−232o*FSHBLuc*-V) was produced using the Adeno-X™ Expression System I since Clonetech system II was discontinued. The differences between System I and II were: System I was not designed for promoterless use so the CMV promoter had to be removed on the shuttle plasmid. Moreover, System I cannot use the efficient recombinase technique. As with system II, the −232o*FSHBLuc* construct was amplified using the Advantage-HF 2 PCR Kit (Clontech) with the following primers:

5′-ttgattattgactagtcaaggtaaaggagtgggtgg-3′ (−232 forward) and.

5′-ccgtttaaacgctagctcttatcatgtctgctcgaa-3′ (−232 reverse).

The pShuttle2 plasmid was cut with SpeI and NheI (Promega) to remove the CMV promoter. The amplified sequence was infused into pShuttle2 using the Fusion Advantage PCR Cloning Kit (Clontech). Finally, the sequence was cut out and ligated into the adenoviral backbone according to the protocol. This insertion of the foreign DNA was confirmed by PCR using the Adeno-X™ PCR screening primer set (Clontech, Mountain View, CA).

When all the three adenoviral constructs were made, they were transfected into a batch of HEK293 cells fresh from ATCC which were cultured the same as LβT2 cells (see above). The viruses were amplified and harvested according to the Clonetech protocol. After 3–4 generations of amplification, the virus titers reached 10^9^ ifu/ml.

### Virus Tittering

Virus titers were determined using the Adeno-X™ Rapid Titer Kit (Clontech, Mountain View, CA). All three viral constructs were adjusted o the same titer of 10^8^ ifu/ml.

### Infecting Primary Cell Cultures

Primary cell culture was performed as described above. The infection was with the MOI of ∼100 ifu/cell in a volume of 10ul.

### Cellular mRNA Extraction and Real Time-rtPCR

Cellular mRNA was extracted using TRI REAGENT (MRC.Inc., Cincinnati, OH). Then mRNA was converted into cDNA using the iScriptTM cDNA Synthesis Kit (BIO-RAD, Hercules, CA). For real time PCR, the setting for each well in the 96-well plate was: 1ul cDNA, 15ul Universal PCR Master Mix (TaqMan, Carlsbad, CA), 13.7ul dH_2_O, 0.1ul of each primer and probe (IDT, Newark, NJ). The primers and probes for 18S, mouse prolactin cDNA, and mouse FSHB cDNA were as follows:

the primers and probe for 18S were

5′-gaaactgcgaatggctcattaa-3′ (forward),

5′-gaatcaccacagttatccaagtagga-3′ (reverse),

and 5′−/56FAM/atggttcctttggtcgctcgctcc/3BHQ_1/−3′ (probe);

for mouse prolactin they were.

5′-tctcaaggtcctgaggtgccaaat-3′ (forward),

5′-caattgcacccaagcatgcactga-3′ (reverse) and

5′−/56-FAM/acaactgctaaacccacattcagtcca/3BHQ_1/−3′ (probe);

for mouse *FSHB* they were

5′-agagaaggaagagtgccgtttctg -3′ (forward),

5′- acatactttctgggtattgggccg-3′ (reverse),

and 5′−/56FAM/atcaataccacttggtgtgcgggcta/3BHQ_1/−3′ (probe);

for the H2Kk they were

5′-agacaaggcagctgtctacggaaa-3′ (forward),

5′-gcagattgctctccagcaacagaa-3′ (reverse) and.

5′−/56 FAM/agcatccacagttaccaagtgccc/3BHQ_1/−3′ (probe).

The PCR cycle setting was 50C for 2 minutes, 95C for 10 minutes, then 95C for 15 seconds for 40 cycles followed by 60 C for 1 minute.

### Statistical Analysis

All experiments were performed either in triplicate or quadruplicate and repeated 3 times or more. The data in Table 1 represent means ± sems. Where there are multiple comparisons, data were analyzed using one-way ANOVA with Tukey’s multiple comparison test according to the Prism version 4 (GraphPad Software, Inc., San Diego CA.).

**Table 1:** Expression of LS and LO o*FSHBLuc* transgenes in mouse tissues.

Founder	Sex	Pituitary	Brain	Lung	Liver	Heart	Spleen	Gonad
LS 41716	F	1407±729	7±2	1±0.4	0	0	2±1	4±1
LS 41721	M	129±29	89±23	0	0	0	0	3±1
LS 41756	M	31±7	1±0.4	0	2±1	0	0	0
LO 41760	F	69	0	0	0	0	0	1
LO 41765	F	263	1	0	0	0	0	0
LO 41774	F	206	73	0	0	0	0	0

Tissues were harvested from mice at least 7 weeks old and lysates were assayed for luciferase activity and protein. Values are luciferase activity expressed as relative light units (RLU)×10^−4^/mg/protein. Values for the LS lines represent the mean ± SEM for three animals. LO data are from one mouse each.

## Results

### Specific Pituitary Expression of−2339o*FSHBLuc* and −1866o*FSHBLuc* in Transgenic Mice

**Figure 1:**
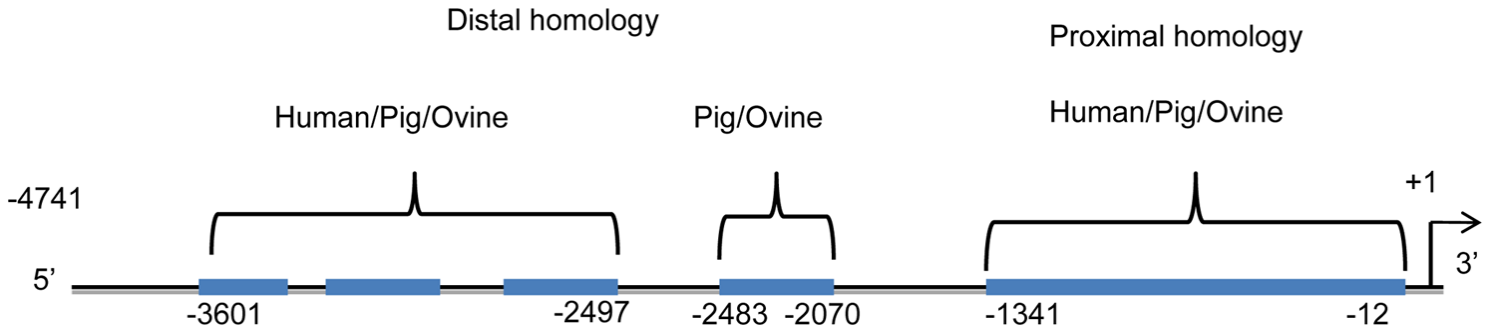
Regions of the ovine FSHB promoter with≥75% sequence homology to porcine and/or human 5′ promoter regions. 4.7 kb of ovine *FSHB* promoter was aligned with 10 kb of human (Ensembl transcript ID: ENST0000025122) and 5.7 kb of porcine (NCBI Acc# D00621.1) *FSHB* promoters using BLAST 2. Regions between −3.6 and −2.5 kb of the ovine *FSHB* promoter are shown that correspond to similar sequences in the human (−5 to −6.1 kb) and porcine (−4.6 to −5.7 kb) *FSHB* promoters. A fourth region of high homology between porcine and ovine *FSHB* promoters was found between −2.48 kb and −2.07 kb.

Previous research showed that −4741 bp of ovine *FSHB* promoter directs robust expression of the luciferase reporter gene specifically to the pituitary, but −750 bp or −215 bp of the same promoter cannot generate any expression [10]. Genomic sequence comparisons of human, pig and sheep *FSHB* promoters found unique promoter regions that show ≥75% homology in all three species in 1.1 kb of the distal promoter (Figure 1). This region contains three sub-domains of ∼100–200 bp each and resides several kb upstream of the *FSHB* start site. High homology was also observed between the ovine and porcine promoters between −2483 bp and 2070 bp. Therefore, computer analysis showed that the porcine, ovine and human *FSHB* promoters contained high homology regions distal to −2339 bp and −1866 bp (Figure 1).

To determine if the highlighted distal regions in Figure 1 are critical for *FSHB* expression, two transgenic mouse lines were produced that contained 2339 bp of 5′ promoter (LO) and 1866 bp (LS). The LO promoter lacks 3 of the upstream high homology sections whereas LS lacks all 4 distal high homology sections. Three founder lines were produced for each LO and LS construct. All founder lines were verified to contain the correct construct using PCR and all founders were fertile with a normal transmission frequency. Pituitaries and other tissues from the homozygous offspring of all founder lines were analyzed for luciferase activity (Table 1). The results show that both the LO and LS transgenic lines expressed luciferase primarily in the pituitary at a level near that produced by the −4741o*FSHBLuc* transgene previously studied [10]. In most cases expression in the pituitary was >100× that in any other tissues except occasionally in brain tissue. Even with −4741o*FSHLuc*, there was occasional high expression in the frontal lobe of the brain.

**Figure 2:**
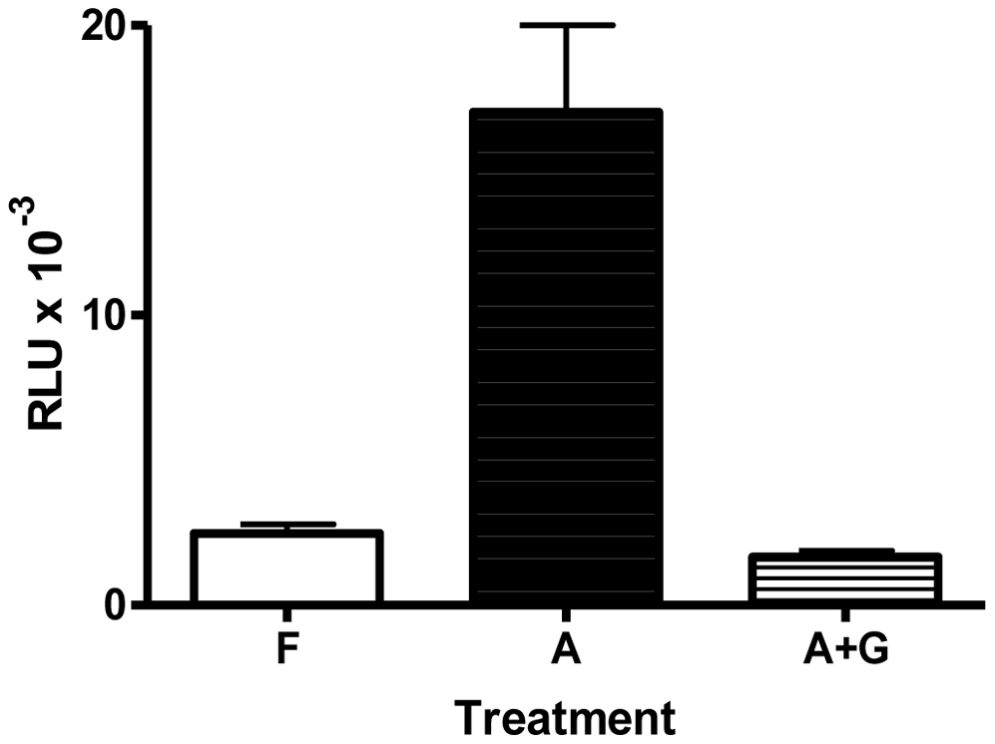
Hormonal regulation of LS-o***FSHBLuc*** **in dispersed pituitary cell culture.** Pituitary cells from transgenic mice expressing LS-o*FSHβLuc* were dispersed and plated at a density of 30,000/well. After 2 days, cells were treated with 250 ng/ml of follistatin (F; basal expression), 50 ng/ml of activin (A; maximal expression) or 50 ng/ml of activin +100 nM GnRH (A+G; GnRH-regulated expression) for 20 h. Cell lysates were assayed for luciferase activity. Values are luciferase activity expressed as relative light units (RLU) and represent the mean ± sem for three independent experiments each performed in triplicate.

To show that the shortest transgene (LS) was regulated by activin and GnRH the same as −4741o*FSHBLuc* [9], [10], dispersed pituitary cultures were produced from the offspring of founder 41716 (see Table 1). The results from these cultures are shown in Figure 2. Since mouse pituitary cultures produce activin from paracrine and/or autocrine sources, cultures were treated with follistatin to incapacitate culture-made activin so gonadotropes produced “basal levels of luciferase.” Activin produced “maximal levels of luciferase” expression (∼7.5-fold induction of luciferase activity) that was also observed for the −4741o*FSHBLuc* transgene [10] and, finally, 100 nM GnRH negatively regulated luciferase as expected from previous studies [9], [10] (Figure 2). In fact, 8 different transgenic o*FHSBLuc* constructs were produced and tested in vivo. Each lacked different combinations of the distal homology region determined to be critical using computer analysis. All constructs expressed specifically in pituitaries and all were regulated by follistatin, activin, and GnRH the same as LS or −4741o*FSHBLuc* (data not shown).

### Comparison of Plasmid and Adenoviral o*FSHBLuc* Constructs in LβT2 Cells

Adenovirus constructs were used to infect primary pituitary cells since plasmid transfection was ineffective. One criterion for judging if adenovirus constructs behaved like plasmid constructs was to compare their expression and regulation in LβT2 cells [16]. Three plasmid constructs were used: −195o*FSHBLuc*, −748o*FSHBLuc* and −2932o*FSHBLuc* [15]. Three similar viral constructs were produced and used: −232o*FSHBLuc*-V, −750o*FSHBLuc*-V and −2871o*FSHBLuc*-V where the “V” designates a viral construct. Although the adenoviral and plasmid constructs were slightly different in lengths due to the use of different cloning sites, both short constructs contained complete enhancer sequences associated with activin induction (and GnRH inhibition) [2]–[5]. The mid-sized constructs (−750o*FSHBLuc*) is the longest construct we have used in transgenic mice that did not express [10]. Finally, the longest construct (−2871o*FSHBLuc*) contained all the sequences needed for robust transgenic expression in mice.

**Figure 3:**
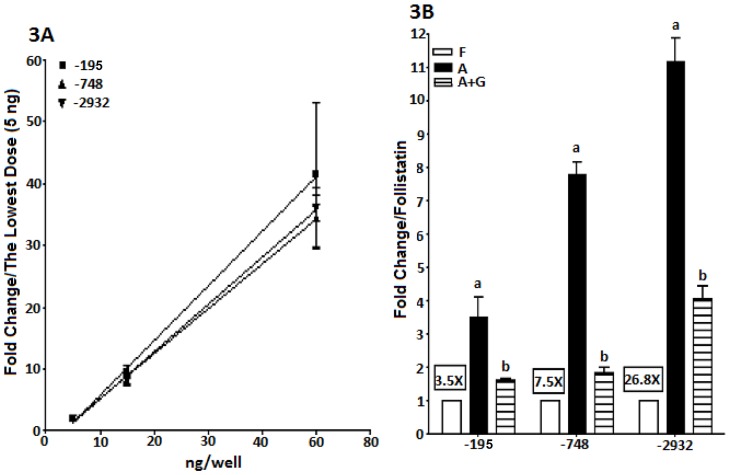
A: Dose-response of plasmid transfection in LβT2 cells. LβT2 cells (40,000 cells/well) were plated and transiently transfected with −195o*FSHBLuc*, −748o*FSHBLuc* or −2932o*FSHBLuc*. Different amounts of DNA (5, 15 or 50 ng/well) were tested. After a 12 hour transfection, cells were treated with activin (50 ng/ml) for 24 h, and then assayed for luciferase activity. For each plasmid, data from different doses were normalized to the activity of the lowest dose. Figure 3B: Hormonal regulation of LβT2 cells transfected with plasmids. LβT2 cells transfected with the 3 plasmid constructs at 50 ng/well (see Fig. 3A) were treated with follistatin (F; 250 ng/ml; basal expression), activin (A; 50 ng/ml; maximal expression) or activin (50 ng/ml)+GnRH (100 nM) (A+G; GnRH-regulated expression) for 24 hours after a 12 hour transfection, and then assayed for luciferase activity. The framed text indicates activin induction for each plasmid construct. For each plasmid, all the data were normalized to the basal activity (follistatin treatment). Different letters indicate significant differences in the means (P<0.05).

**Figure 4:**
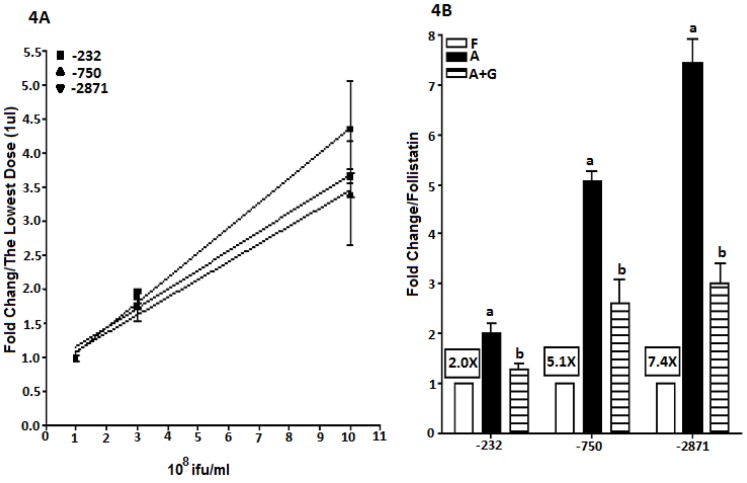
A: Dose-response for viral infection in LβT2 cells. LβT2 cells (40,000 cells/well) were plated and infected with −232o*FSHBLuc*-V, −750o*FSHBLuc*-V or −2871o*FSHBLuc*-V. Different amounts of viral constructs (1, 3 and 10 ul/well of 10^8^ ifu/ml) were tested. After a 12 hour infection, cells were treated with activin (A; 50 ng/ml) for 24 hours, and then assayed for luciferase activity. For each viral vector, data from different doses were normalized to the activity of the lowest dose. Figure 4B: Hormonal regulation of the LβT2 cells infected with viral constructs. LβT2 cells infected with 10 ul of 232o*FSHBLuc*-V, −750o*FSHBLuc*-V or −2871o*FSHBLuc*-V (see Fig. 4A) were treated with follistatin (F; 250 ng/ml; basal expression), activin (A; 50 ng/ml; maximal expression) or activin (50 ng/ml)+GnRH (A+G; 100 nM) (A+G; GnRH-regulated expression) respectively for 24 hours after a 12 hour infection, and then assayed for luciferase activity. The framed text indicates activin induction for each viral construct. For each viral construct, data from different doses were normalized to the activity of the lowest dose. Different letters indicate significant differences of means (P<0.05).

Plasmid and viral constructs were first compared with each other in LβT2 cells using dose-response experiments at three concentrations of plasmid (5 ng, 15 ng and 50 ng) or adenovirus (1 ul, 3 ul and 10 ul of each adenovirus with a titer of 10^8^ifu/ml). Each construct was used to express luciferase in 40,000 LβT2 cells and then the cells were treated with 50 ng/ml activin for 24 hours. All plasmid and viral constructs produced linear dose-responses with luciferase expression being similar for both plasmids and viruses (Figures 3A and 4A).

Since the dose-responses for activin induction (maximal activities) were linear (Figs. 3A and 4A) it was legitimate to compare the effects of follistatin, activin and GnRH plus activin using the highest levels of plasmid or virus shown in Figs. 3A/4A. Again 40,000 cells were treated with plasmid or virus and then cells were treated with follistatin, (250 ng/ml), activin (50 ng/ml) or activin plus GnRH (100 nM) for 24 hours. In all cases the plasmids and viruses behaved alike (see 3B/4B). Follistatin-treated cells produced “basal activity,” activin-treated cultures produced “maximal activity” and GnRH treatment down-regulated activin-induced expression as expected.

**Figure 5:**
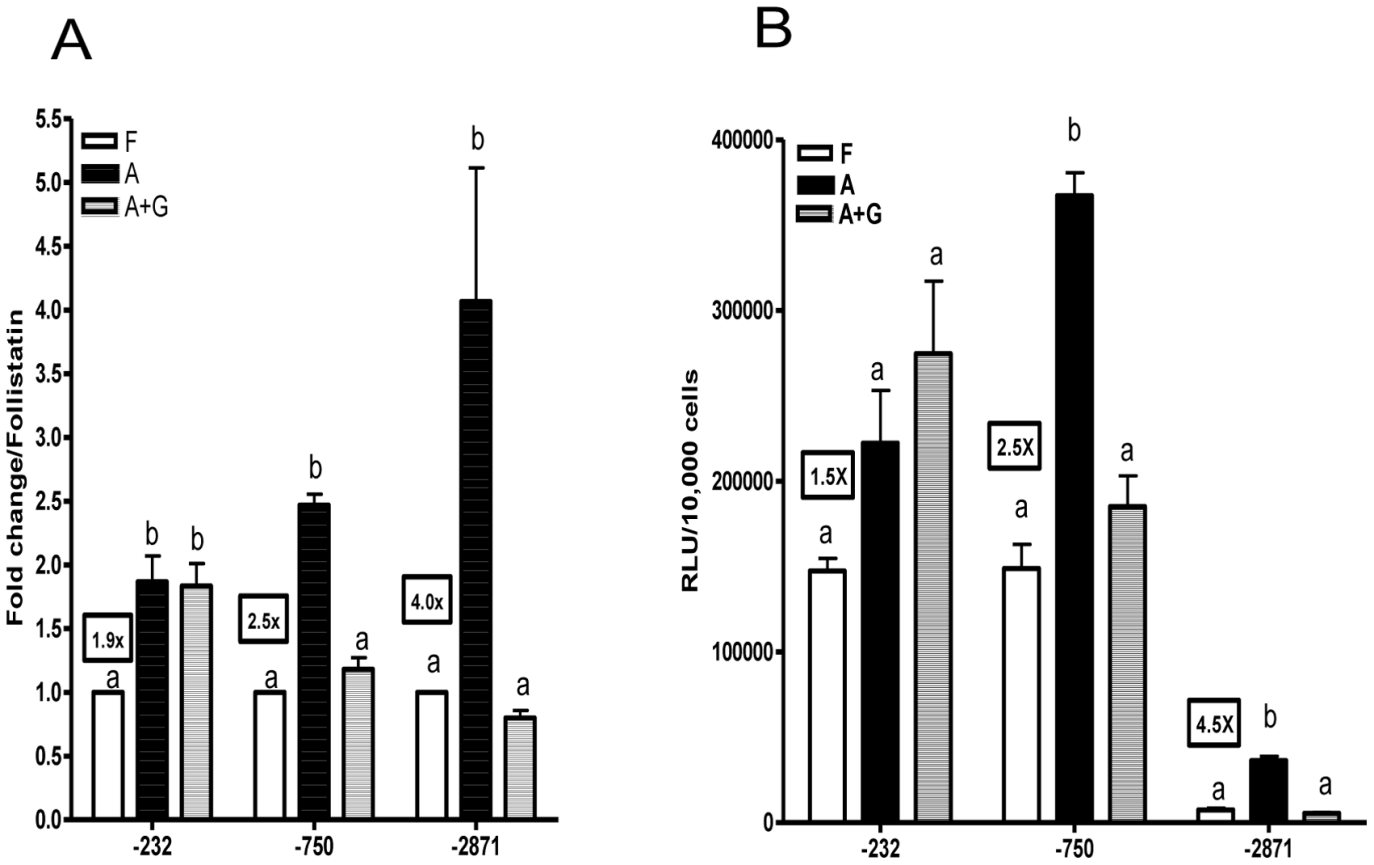
Adenoviral infection of purified gonadotropes. Primary gonadotropes (10,000 cells/well) were plated and infected with −232o*FSHBLuc*-V, −750o*FSHBLuc*-V or −2871o*FSHBLuc*-V. After a 24 hour infection, cells were treated with follistatin (F; 250 ng/ml; basal expression), activin (A; 50 ng/ml; maximal expression) or activin (50 ng/ml)+GnRH (100 nM) (A+G; GnRH-regulated expression) respectively for 48 hours. Experiments were performed in quadruplicate and repeated 3 times. **Figure 5A** shows combined results for three individual experiments. Follistatin results (basal expression) were set a value of 1. The framed text indicates activin induction for each viral construct. Bars with different letters are significantly different (P<0.05). **Figure 5B** shows non-normalized data from one of the three independent experimental replicates. The framed text indicates activin induction for each viral construct. Bars with different letters are significantly different (P<0.05).

**Figure 6:**
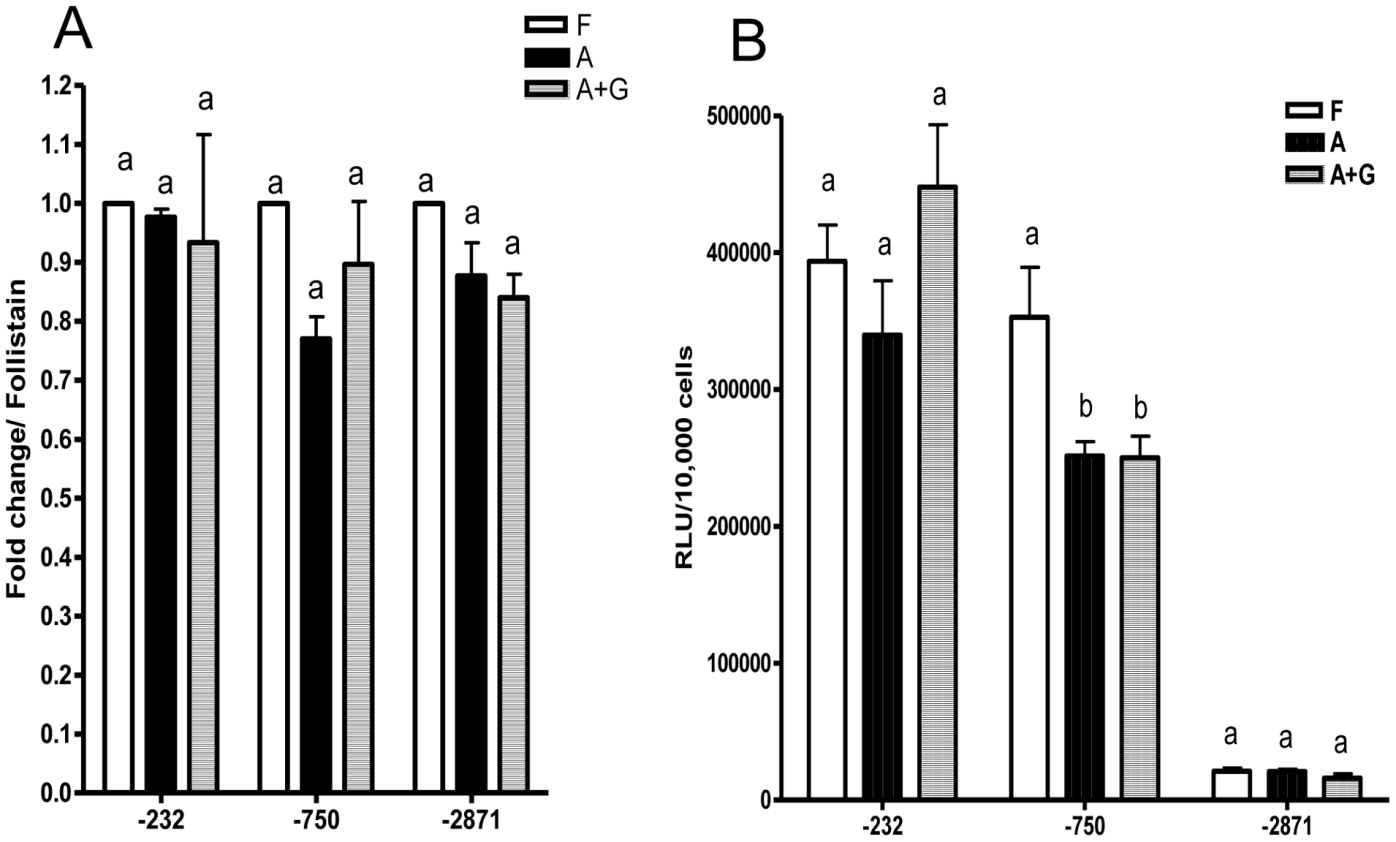
Adenoviral infection of non-gonadotropes (flow through cells). Non-gonadotropes (10,000 cells/well) were plated and infected with −232o*FSHBLuc*-V, −750o*FSHBLuc*-V or −2871o*FSHBLuc*-V. After a 24 hour infection, cells were treated with follistatin (F; 250 ng/ml; basal expression), activin (A; 50 ng/ml; maximal expression) or activin (50 ng/ml)+GnRH (100 nM) (A+G; GnRH regulated expression) for 48 hours. Experiments were performed in quadruplicate and repeated 3 times. **Figure 6A** shows combined results for three individual experiments. Follistatin results (basal expression) were adjusted to 1. Different letters indicate significantly different means (P<0.05). **Figure 6B** represents data from one representative replicate. Different letters indicate significantly different means (P<0.05).

Both adenoviral and plasmid constructs behaved alike in LβT2 cells as shown in Figs. 3 and 4. The data in Figs. 3B and 4B are almost superimposable. This observation allowed us to continue our studies in primary pituitary cells where any differences in behavior would be construed as being due to special factors in the primary cells that are not present in LβT2 cells (Figs. 5 & 6).

It should be noted that the data in Figs. 3B and 4B show that basal expression decreased and induction by activin increased with construct size. This effect had been seen many times in past studies which led to the theory that the longer 5′ promoter constructs contained negative control elements that depressed basal expression thereby increasing the relative effect of activin induction. Ultimately this effect was shown to be an artifact and was, in fact, due only to changes in plasmid size rather than promoter content. It was interesting to note the same artifact with the adenoviral constructs [15].

### Purifying Primary Gonadotropes from H2Kk Transgenic Mice and Testing their Purity

Mouse pituitaries from transgenic mice expressing H2Kk on the surface of their pituitary gonadotropes were dispersed and purified by magnetic immunomicrobead technology as reported earlier [12]. Both primary gonadotropes and flow through cells (non-gonadotropes) went through two cycles of purification, were plated at 10,000 cells/well and then treated with activin A (50 ng/ml) for 48 hours as reported [12].

Gonadotrope and non-gonaotrope populations were assayed by real-time rtPCR for murine prolactin and FSHB mRNAs plus 18s RNA (internal standard for both PCR assays). Lactotropes comprise 30–40% of mouse pituitary cells and FSHB comes solely from gonadotropes which comprise ∼5% mouse pituitary cells [12]. Based on the percent decrease in prolactin mRNA between flow through cells and the gonadotrope fraction, the purity of gonadotropes was calculated to be 96%, 89% and 91% for three separate preparations and the level of FSHB mRNA was 30- to 60-fold higher in gonadotrope fractions compared to non-gonadotropes. These preparations were used to generate the data in Figures 5 and 6.

### Expression and Regulation of viral o*FSHBLuc*-V Constructs in Purified Primary Gonadotropes

The data in Figure 5A are normalized composite results from three separate experiments (from the three preparations described above) showing expression of the three viral constructs in purified gonadotropes and comparing the results of follistatin (basal expression), activin (maximal expression) and activin plus GnRH treatments (GnRH-regulated expression). The data in Figure 5B are non-normalized data from a single experiment (from preparation 3 above, 91% pure gonadotropes). The data in Figure 5B show clearly that −232o*FSHBLuc*-V and −750o*FSHBLuc*-V are expressed much better than the longer −2871o*FSHBLuc*-V construct. These same results have been observed in LβT2 cells [16]. If primary cells had behaved like cells in vivo, they would not have expressed these constructs.

As expected GnRH inhibited the −750o*FSHBLuc*-V and −2871o*FSHBLuc*-V constructs, but surprisingly it did not inhibit expression of −232o*FSHBLuc*-V. This phenomenon was not observed in LβT2 cells and indicates a significant difference between regulation by GnRH in primary and LβT2 transformed gonadotropes.

### Expression and Regulation of Viral o*FSHBLuc*-V Constructs in Non-gonadotropes

Figure 6A shows normalized composite data for results from three separate experiments showing expression of the three viral constructs in non-gonadotropes. Figure 6B is a representative single experiment showing non-normalized data using non-gonadotropes. In both figures (A&B) it is clear that there is considerable expression of all ovine *FSHBLuc*-V constructs in non-gonadotropes. There should not have been any expression in these cells if proper cell-specific targeting had occurred. A statistical comparison of the data in Figures 5 and 6 indicates that basal expression of o*FSHBLuc*-V constructs is not significantly different in gonadotropes compared to non-gonadotropes. Finally, it should be noted that neither activin nor GnRH (100 nM) had any significant effect on expression of any of the o*FSHBLuc*-V constructs in non-gonadotropes as expected.

## Discussion

### Cell Specific Expression– the Transgenic Approach

Prior to this study, 12 founder mice were produced that contained 4741 bp of ovine *FSHB* promoter linked to the luciferase gene and all but two expressed high levels of luciferase (1–10 million RLU/mg protein) in the pituitary [10]. This was at least 100× the activity in all other tissues tested, except occasionally the frontal lobe of the mouse brain [6], [10]. Considering that only 1/20^th^ (∼5%) of pituitary cells are gonadotropes and they are the only pituitary cells producing luciferase [17], the specificity of expression for −4741o*FSHBLuc* in gonadotropes versus non-gonadotropes is calculated to be 20×100 = 2,000∶1.

In this prior study, 7 transgenic founders containing −215o*FSHBLuc* and 3 transgenic founders containing −750o*FSHBLuc* were also produced and none expressed luciferase in any tissue [10]. These data indicated that sequences important for gonadotrope-specific expression of o*FSHB in vivo* exist between −750 bp and −4741 bp of the ovine *FSHB* promoter. One of the −4741o*FSHBLuc* transgenes that did not express in vivo had a deletion from −2755 bp to −3275 bp suggesting that important elements within this sequence might be especially important for gonadotrope expression. Figure 1 of this paper shows that the ovine *FSHB* promoter contains three sequences between −2755 bp and −3275 bp that have high homology to similar sequences on the human and porcine *FSHB* promoters. The 5′ sequences of the human promoter that are homologous to the ovine sequences are so far upstream of the transcription start site (−5 to −6.1 kb) they have never been included or tested for importance in any human *FSHBLuc* transgene. The concept that these conserved sequences might help control *FSHB* expression in many vertebrates needed to be tested. Therefore, we produced transgenics that carried ovine *FSHBLuc* constructs missing the conserved homologous regions, −2361o*FSHBLuc* (LO) and −1866o*FSHBLuc* (LS). As noted in results, we created 8 different constructs longer than LO and all expressed at least as well as LO and LS (see Table 1); all were regulated like the LS construct (−1866o*FSHBLuc*) by follistatin, activin and activin plus GnRH as well as LS (Fig. 2) (data not shown).

We show here that the LS transgene (−1866o*FSBHBLuc*) was expressed even better than the LO transgene (−2361o*FSHBLuc*) and equally well as −4741o*FSHBLuc* previously reported [10]. This means that no sequence 5′ to −1866 bp is needed for o*FSHB* expression. Therefore, the critical 5′ sequences lie between −1866 bp and −750 bp for gonadotrope-specific expression of ovine *FSHB*. Since the porcine promoter sequence is different from the ovine sequence in this region and the human *FSHB* gene requires 3′ sequences for expression, it may be that unique requirements exist for the expression of many vertebrate *FSHB* genes. This later scenario seems highly unlikely, however, for a gene so important for the preservation of all vertebrates.

### Cell Specific Expression– the Primary Gonadotrope Approach

Since the homology approach did not locate 5′ distal promoter sequences needed for gonadotrope-specific expression of ovine *FSHB*, and since LβT2 cells have not been useful either, we turned to primary gonadotropes believing they might contain the information needed to create gonadotrope specific expression. The following 3 adenoviral constructs were made for expression in primary gonadotropes (and non-gonadotropes) to study gonadotrope specific expression of ovine *FSHB*: −232o*FSHBLuc*-V, −750o*FSHBLuc*-V, and −2871o*FSHBLuc*-V.

The shortest, −232o*FSHBLuc*-V, contained sequences previously known to be important for gonadotrope-specific regulated expression [2]–[5] and was regulated just like its similar plasmid construct in LβT2 cells by follistatin (basal activity), activin (maximal induction) and GnRH plus activin (negative regulation by GnRH). Surprisingly, this construct was not inhibited by GnRH when expressed in primary gonadotropes and treated with activin (compare Figs. 4B & 5B). This indicated that primary gonadotropes do contain regulatory mechanisms not found in LβT2 cells. It also suggests there are sequences between −750/−232 bp that mediate negative GnRH regulation in vivo that work differently from the mechanisms that occur in LβT2 cells. In addition, GnRH completely inhibited activin-induced expression of −750o*FSHBluc* and −2871*FSHBLuc* in primary gonadotropes (Fig. 5A & 5B) as it did with the LS transgene (−1866o*FSHBLuc*) in primary pituitary cultures (Fig. 2). Full inhibition by GnRH is has never been observed in LβT2 cell cultures (see Figs. 3 & 4). This area of research is open to further investigation.

Aside from the differences noted above for GnRH inhibition of activin-induced *FSHB* expression, there were no significant differences observed between basal or regulated expression of either the plasmid constructs or adenoviral constructs when expressed in LβT2 cells or primary gonadotropes. Therefore, we believe that the use of either primary gonadotropes or LβT2 cells is not helpful for learning about sequences in the distal 5′ promoter of the ovine FSHB gene needed for expression in vivo.

It should be noted that none of the adenoviral constructs were regulated by follistatin, activin or GnRH in non-gonadotropes in the pituitary. The lack of response to GnRH was expected since non-gonadotropes have no GnRH receptors, but some or many pituitary cell types might have responded to activin. Either most do not carry the activin receptor or do not have signaling mechanisms to induce the o*FSHBLuc* constructs.

The data in Figure 5B show that expression of o*FSHBLuc* constructs decreases with promoter length as activin induction increases. This might suggest there are negative control elements in the distal 5′ promoter of o*FSHB* that decrease basal expression that accentuates induction by activin. It should be noted, however, that the same phenomenon occurs in non-gonadotropes (Fig. 6B). These cells have no natural regulatory mechanism for expressing o*FSHB* so this phenomenon does not reflect normal o*FSHB* expression. As noted under results, this phenomenon has been thoroughly studied and the effect is an artifact created by a decrease in plasmid (or adenoviral) size [15]. It is an interesting artifact, but not physiologically relevant.

IN CONCLUSION, it has been shown here that 5′ distal promoter sequences between −1866/−750 bp of the ovine *FSHB* gene are necessary for *FSHB* expression in vivo. Unfortunately, neither primary gonadotropes nor LβT2 cells seem suitable for identifying these important sequences. It has been shown, however, that primary gonadotropes can be used to further investigate o*FSHB* promoter sequences between −750/−232 bp that negatively regulate activin-induced expression in vivo. Finally, it is worth noting that transgenic studies could efficiently and rapidly identify the critical sequences needed for gonadotrope-specific expression given the information presented herein.
